# Genetic and Lifestyle Risk Factors of Metabolic Dysfunction-Associated Fatty Liver Disease and Its Relationship with Premature Coronary Artery Disease: A Study on the Pars Cohort

**DOI:** 10.34172/aim.2024.36

**Published:** 2024-05-01

**Authors:** Amir Anushiravani, Maryam rayatpisheh, Amir Kasaeian, Iman Menbari Oskouie

**Affiliations:** ^1^Digestive Diseases Research Center, Digestive Diseases Research Institute, Shariati Hospital, Tehran University of Medical Sciences, Tehran, Iran; ^2^Digestive Oncology Research Center, Digestive Diseases Research Institute, Shariati Hospital, Tehran University of Medical Sciences, Tehran, Iran; ^3^Research Center for Chronic Inflammatory Diseases, Shariati Hospital, Tehran University of Medical Sciences, Tehran, Iran; ^4^Clinical Research Development Unit, Shariati Hospital, Tehran University of Medical Sciences, Tehran, Iran; ^5^Pediatric Urology and Regenerative Medicine Research Center, Gene, Cell & Tissue Research Institute, Tehran University of Medical Sciences, Tehran, Iran; ^6^Urology Research Center, Tehran University of Medical Sciences, Tehran, Iran; ^7^Center for Orthopedic Trans-Disciplinary Applied Research, Tehran University of Medical Sciences, Tehran, Iran

**Keywords:** Cardiovascular diseases, Genetics, Non-alcoholic fatty liver disease, Metabolic syndrome, Obesity

## Abstract

**Background:**

The main objective of this study is to identify the risk factors of metabolic dysfunction-associated fatty liver disease (MAFLD) in coronary artery disease (CAD) patients.

**Methods:**

The present retrospective cohort study is part of the Pars Cohort Study (PCS). The participants were categorized as having MAFLD or not. The pattern of independent variables in patients was compared with those who did not have MAFLD. All variables were retained in the multivariable logistic regression model.

**Results:**

Totally, 1862 participants with CAD were enrolled in this study. MAFLD was diagnosed in 647 (40.1%) participants. Gender, diabetes, hypertension, tobacco, opium, alcohol, age, weight, waist circumference, cholesterol, HDL, triglyceride, aspartate aminotransferase (AST), and alanine aminotransferase (ALT) were significantly different in MAFLD and non-MAFLD patients. Also, the results of multivariable logistic regression show male gender (OR=0.651, 95% CI: 0.470‒0.902, *P* value=0.01) and opium consumption (OR=0.563, 95% CI: 0.328‒0.968, *P* value<0.001) to be negative risk factors of MAFLD occurrence in CAD patients. Having diabetes (OR=2.414, 95% CI: 1.740-3.349, *P* value<0.001), high waist circumference (OR=1.078, 95% CI: 1.055‒1.102, *P* value<0.01), high triglyceride (OR=1.005, 95% CI: 1.001‒1.008, *P* value=0.006), and high ALT (OR=1.039, 95% CI: 1.026‒1.051, *P* value<0.01) were positive risk factors of MAFLD in CAD patients.

**Conclusion:**

Our study found that consuming opium decreases the likelihood of MAFLD in CAD patients, since these patients have decreased appetite and lower body mass index (BMI). On the other hand, female gender, having diabetes, high waist circumference, high triglyceride levels, and high ALT levels increase the probability of MAFLD in CAD patients.

## Introduction

 Hepatic fibrosis and steatosis are important clinical conditions that can lead to liver cirrhosis, portal hypertension, and hepatocellular carcinoma. The presence of hepatosteatosis in the absence of alcohol consumption is called metabolic dysfunction-associated fatty liver disease (MAFLD).^1^ MAFLD is a significant cause of chronic liver disease and is a hepatic manifestation of metabolic syndrome (MetS), which is a major risk factor for cardiovascular disease (CVD) and type 2 diabetes.^2,3^

 The prevalence of MAFLD has increased globally, making it a major public health concern. However, determining the actual incidence and prevalence of MAFLD can be difficult due to the diversity in the definition of the disease and lack of standard diagnostic methods. Approximately 25% of the world’s population suffers from MAFLD, with 5% experiencing nonalcoholic steatohepatitis (NASH), and approximately 20% developing liver cirrhosis.^4^ In Asian countries, the prevalence of MAFLD varies from 12.2% in the Philippines to 17.2% in South China. It is expected that with the increase in the prevalence of obesity, diabetes, and MetS in Asian countries, MAFLD incidence will continue to rise in the current decade and in the future.^5,6^ In studies conducted in Iran, the prevalence of MAFLD has been reported to be about 35%.^7-11^ Contrary to previous studies that found higher prevalence in women, recent studies suggest that MAFLD is independent of sex, age, and socioeconomic status.^12^

 The main mechanism for MAFLD is insulin resistance, and its main risk factors are obesity, diabetes, and hyperlipidemia.^13^ Diabetes mellitus is one of the most common metabolic diseases in the world, and it is the main metabolic cause of MAFLD. In Iran, obesity and MetS are predictors of increasing fatty liver disease, so it is expected that the prevalence of MAFLD/NASH and its related complications will increase in the future.^14^ Additionally, the presence of MAFLD in diabetic patients is a risk factor for increasing CVDs. Over the past decade, it has become increasingly clear that MAFLD is strongly associated with increased risk of CVDs, which are the leading cause of death among MAFLD patients.^15^

 CVDs are one of the leading causes of death in various societies, with heart attacks being particularly significant. Despite a decline in prevalence over the past few decades, heart attacks remain a major cause of morbidity and mortality in both developed and developing countries. Premature coronary artery disease (CAD), an atherosclerotic condition that affects individuals under the age of 45 (and up to 65), is an early manifestation of this condition.^16^ Despite existing evidence that MAFLD is also associated with other chronic diseases, the negative effect of MAFLD on CVD risk is increasing due to the obesity/MetS epidemic in the general population.^17^

 Genetics plays a crucial role in the development of MAFLD. The heritability of hepatic fibrosis and steatosis has been studied in limited populations and the genetic factors associated with these conditions are not well understood.^18^ There is a need to identify the genetic factors associated with hepatic fibrosis and steatosis, particularly in adult populations, and to determine their contribution to the development of these conditions. The identification of genetic factors associated with hepatic fibrosis and steatosis may provide insights into the underlying mechanisms of these conditions and may lead to the development of new therapeutic strategies.^19,20^

 The main objective of this study is to identify the risk factors of MAFLD in CAD patients. Through our study, we hope to uncover new insights to identify potential targets for prevention and treatment.

## Materials and Methods

###  Cohort Study and Participants

 The present retrospective cohort study is part of Pars Cohort Study (PCS). PCS was initiated in 2012 across the Valashahr area, a countryside region situated in southern Iran. The Valashahr region is home to around 40 000 residents, mainly of Persian or Turk ethnicity. Individuals who showed unwillingness to participate in the current research or were not permanent residents were not included. Additional information regarding PCS has been discussed in another study.^21^

 A total of 1862 CAD participants aged more than 50 years were enrolled in the study. CAD was defined as a cardiovascular event, such as myocardial infarction, cerebrovascular accident, and having a percutaneous coronary intervention (PCI) or coronary artery bypass graft (CABG). Data were collected using a structured questionnaire.

###  Study Outcome

 The participants were categorized as having MAFLD or not. The pattern of independent variables in patients was compared with those who did not have MAFLD. Patients with MAFLD were included if they met the criteria for diagnosis of MAFLD according to the guidelines of the American Association for the Study of Liver Diseases.^22^ Participants with a history of alcohol consumption, liver cirrhosis, or other liver diseases were excluded from the study.

###  Variables Measurement

 Although PCS involves a substantial number of variables (over 180 in total), a limited number of variables were chosen based on self-report, physical examinations, and biological samples. The conceptual framework used to select independent variables considered biological factors, data on MAFLD’s prevalence, and detection and evaluation of any possible variables linked to this disease. Skilled interviewers used a general questionnaire to gather the necessary information for each individual. The questionnaire inquires about demographic characteristics such as gender, marital status, age, race, education level, and medical status. It also includes self-reported data on opium, cigarette, other types of tobacco, and alcohol consumption. Height, weight, waist circumference, and hip diameter were measured for each individual. The blood pressure (BP) of each participant was assessed by the same examiner utilizing a mercury sphygmomanometer while seated, following a 5-minute relaxation period. Two BP measurements were taken for each arm, with a ten-minute interval between them. The average of these readings was recorded to determine each participant’s BP. After that, the BP of each individual was measured in the standing position. In addition, we collected 5-mL blood samples to measure fasting blood sugar (FBS), total cholesterol, low-density lipoprotein (LDL), high-density lipoprotein (HDL), triglyceride, alkaline phosphatase (ALP), glycohemoglobin (HbA1c), aspartate aminotransferase (AST), and alanine transaminase (ALT) for each participant.

 The validity and reliability of the instruments used for data collection, including a structured questionnaire and physical examinations, are previously described.^21^

###  Statistical Analysis

 Quantitative variables were described by means ± standard deviation (SD) and median (interquartile range (IQR)). Also, qualitative variables were described by frequencies and percentage. Independent samples *t*test or Mann-Whitney U test were used for comparison of quantitative variables and chi-square and/or Fisher’s exact test were used for comparison of qualitative variables. All variables were retained in the multivariable logistic regression model. *P* values less than 0.05 were considered as statistically significant. Statistical analyses were done using the STATA software version 17.

## Results

 Overall, 1862 participants with CAD were enrolled in this study. There were 953 (51.2%) males and 909 (48.8%) females with a mean age of 58.9 ± 6.76. Of these, 16.3% had diabetes and 45.3% had hypertension. MAFLD was diagnosed in 647 (40.1%) participants, and 411 patients had grade I, 175 patients had grade II, and 61 patients had grade III fatty liver. The prevalence of opium use was 8.6%, tobacco was 18.4%, and alcohol was 5.4%. Other variables are described in Tables 1 and 2.

**Table 1:** Baseline Quantitative Characteristics of the Pars Cohort Study Participants with CVD (N = 1862)

**Variable**	**No.**	**Mean (±SD)**	**Median (IQR)**
Age	1862	58.9 (6.76)	58 (53‒64)
Height	1860	161.7 (9.29)	161.5 (154.5‒169)
Weight	1860	74.01 (13.53)	73 (65‒82.5)
Waist circumference	1860	99.75 (12.32)	100 (92‒108)
Hip circumference	1860	101.5 (8.94)	100 (96‒106)
FBS	1862	110.7 (44.4)	96.7(87.9‒111)
Cholesterol	1862	213.2 (42.7)	211 (185‒239)
HDL	1862	59.1 (15.1)	57 (49‒68)
LDL	1862	122.5 (37.5)	123 (100‒146)
Triglyceride	1862	149.3 (97.1)	127 (93‒180)
ALP	1862	256.6 (91.1)	243 (203‒289)
HbA1c	463	7.11 (2.25)	7.06 (5.87‒8.4)
AST	1862	21.5 (11.9)	19 (16‒24)
ALT	1861	23.5 (17.0)	19 (14‒27)
BP sitting diastolic	1862	80.3 (11.2)	80 (72‒88)
BP sitting systolic	1862	133.8 (22.5)	132 (120‒148)
BP standing diastolic	1857	81.0 (12.2)	80 (72‒90)
BP standing systolic	1858	132.0 (22.9)	130 (118‒146)

CVD, Cardiovascular disease; Circ, Circumference; FBS, Fast blood sugar; HDL, High density lipoprotein; LDL, Low-density lipoprotein; ALP, Alkaline phosphatase; AST, Aspartate aminotransferase; ALT, Alanine transaminase; BP, Blood pressure

**Table 2:** Baseline Categorical Characteristics of the Pars Cohort Study Participants with CVD (N = 1862)

**Variable**	**Subgroup**	**No. (%)**
Gender	Female	909 (48.8%)
	Male	953 (51.2%)
Diabetes	No	1558 (83.7%)
	Yes	304 (16.3%)
Hypertension	No	1019 (54.7%)
	Yes	843 (45.3%)
Fatty liver	No	967 (59.9%)
	Yes	647 (40.1%)
Fatty liver grade	Normal	967 (59.9%)
	Grade I	411 (25.5%)
	Grade II	175 (10.8%)
	Grade III	61 (3.8%)
Tobacco	No	1517 (81.6%)
	Yes	341 (18.4%)
Opium	No	1698 (91.4%)
	Yes	160 (8.6%)
Alcohol	No	1757 (94.6%)
	Yes	101 (5.4%)

CVD, Cardiovascular disease.

 Patients were divided into two groups: MAFLD and non-MAFLD. Table 3 shows the distribution of each variable in these two groups. Gender, diabetes, hypertension, tobacco, opium, alcohol, age, weight, waist circumference, cholesterol, HDL, triglyceride, AST, and ALT were significantly different between MAFLD and non-MAFLD patients. Also, the results of multivariable logistic regression demonstrated in Table 3 show the correlation of variables with the occurrence of MAFLD. Male gender (OR = 0.651, *P *value = 0.01) and opium consumption (OR = 0.563, *P *value < 0.001) were negative risk factors of MAFLD occurrence in CAD patients. Having diabetes (OR = 2.414, *P *value < 0.001), high waist circumference (OR = 1.078, *P *value < 0.01), high triglyceride (OR = 1.005, *P *value = 0.006), and high ALT (OR = 1.039, *P *value < 0.01) were positive risk factors of MAFLD in CAD patients.

**Table 3:** Logistic Regression Analysis of Factors Associated with MAFLD in CVD Patients in PARS Cohort Study

**Variable**	**Subgroup (Unit)**	**No. (%)/Median (IQR)**		***P *** **Value**	**Adjusted Odds Ratio ** **(95% CI)**	***P *** **Value**
		**MAFLD**	**No MAFLD**			
Gender	Female	361 (22.4%)	423 (26.2%)	< 0.001	Reference	0.010*
	Male	286 (17.7%)	544 (33.7%)		0.65 (0.47‒0.90)	
Diabetes	No	467 (28.9%)	882 (54.6%)	< 0.001	Reference	< 0.001*
	Yes	180 (11.1%)	85 (5.3%)		2.41 (1.74‒3.35)	
Hypertension	No	308 (19.1%)	546 (34.0%)	< 0.001	Reference	0.884
	Yes	339 (21.0%)	421 (26.1%)		1.02 (0.80‒1.30)	
Tobacco	No	562 (34.8%)	761 (47.1%)	< 0.001	Reference	0.669
	Yes	84 (5.20%)	205 (12.7%)		0.92 (0.63‒1.34)	
Opium	No	620 (38.5%)	856 (53.1%)	< 0.001	Reference	0.038*
	Yes	26 (1.6%)	110 (6.8%)		0.56 (0.33‒0.97)	
Alcohol	No	616 (38.2%)	913 (56.6%)	0.453	Reference	0.562
	Yes	30 (1.9%)	53 (3.3%)		0.84 (0.47‒1.51)	
age	years	57 (53‒62)	58 (53-64)	0.023	0.99 (0.97‒1.01)	0.180
Weight^a^	Kg	79.44 (13.22)	70.37(12.49)	< 0.001	1.00 (0.98‒1.02)	0.972
Waist circumference^a^	cm	106.05(10.32)	95.56(11.73)	< 0.001	1.08 (1.055‒1.102)	< 0.01*
Cholesterol^a^	mg/dL	216.9(43.5)	211.0(41.5)	0.006	0.99 (0.98‒1.01)	0.805
HDL^a^	mg/dL	56.3(13.6)	60.9(15.2)	< 0.001	0.99 (0.98‒1.01)	0.415
LDL^a^	mg/dL	121.6(39.9)	123.1(35.9)	0.443	1.00 (0.99‒1.01)	0.866
Triglyceride	10 mg/dL	153 (110‒213)	114 (86‒155)	< 0.001	1.50 (1.10‒1.80)	0.006*
ALP	IU/L	245 (205‒297)	239 (200‒283)	0.065	0.99 (0.97‒1.00)	0.074
AST	IU/L	21 (16‒26)	19 (15‒23)	< 0.001	0.99 (0.97‒1.00)	0.119
ALT	IU/L	24 (18‒36)	17 (12‒24)	< 0.001	1.04 (1.03‒1.05)	< 0.01*

MAFLD, Metabolic dysfunction-associated fatty liver disease; CVD, Cardiovascular disease; HDL, High density lipoprotein; LDL, Low density lipoprotein; ALP, Alkaline phosphatase; AST, Aspartate aminotransferase; ALT, Alanine transaminase. ^a^Mean (SD) was reported for these variables due to normal distribution. *Statistically significant.

## Discussion

 MAFLD is currently believed to be the most prevalent liver disease, impacting approximately one billion individuals globally,^23^ and gene-environment interactions determine the phenotypic displays and grade of MAFLD.^24^ MAFLD encompasses an extensive range of liver injury severity, such as simple steatosis, steatohepatitis, fibrosis, and ultimately cirrhosis and hepatocellular carcinoma. Most diagnosed individuals have histological alterations classified as mere steatosis,^25^ with only a minority experiencing chronic inflammation, which indicates disease worsening. Additionally, there is significant evidence of variability among individuals in all aspects of the natural history.^26^ Multiple factors may contribute to the progression from steatosis to NASH and subsequent hepatic fibrosis.^27^

 Studies including familial aggregation and research on twins have provided confirmation suggesting that MAFLD has a heritable element.^28,29^ Interestingly, there may be a genetic predisposition for the occurrence of both steatosis and fibrosis.^30^ There are differences in the susceptibilities of various races for severe disease; Hispanic patients are more sensitive than white patients, while black patients are the least vulnerable.^31,32^ So, one could argue that MAFLD is a multifactorial disorder with unique complexities. Nonetheless, research on the determinants of MAFLD continues with a focus on at-risk individuals

 This historical cohort study aimed to clarify the risk factors for MAFLD occurrence in CAD patients. The results show that being male and consuming opium decrease the chance of MAFLD in CAD patients. Being male reduces the chances of MAFLD by 35% compared to females. Patients who use opium have a 45% lower probability of MAFLD occurrence. This negative association between gender and MAFLD is probably due to the fact that females were more obese and overweight in PCS.

 On the other hand, having diabetes, high waist circumference, high triglyceride levels, and high ALT levels increase the probability of MAFLD in CAD patients. Diabetes increases the chances of MAFLD by 2.4 times. Increasing triglyceride levels and ALT levels increase the probability of MAFLD by 0.5% and 3.9% per unit, respectively.

 MAFLD has become a widespread issue in society, affecting a significant portion of the global community. It is estimated that nearly 25% of people worldwide are affected.^4^ In Asian countries, the prevalence of MAFLD ranges from 12.2% in the Philippines to 17.2% in South China. The increasing rates of obesity, diabetes, and MetS in Asian nations are likely to contribute to a further rise in MAFLD cases in the coming years.^5,6^ Researches conducted in Iran have shown that the prevalence of MAFLD is about 35%.^7-11^ Our investigation found that 40.1% of CAD patients in the Pars cohort were diagnosed with MAFLD, a higher prevalence compared to previous studies.

 A variety of biochemical parameters tended to fluctuate in tandem with the appearance and progression of fatty liver. Our investigation revealed elevated quantities of liver-specific enzymology indices and lipid metabolism factors in individuals with MAFLD. Specifically, we observed increased levels of ALT, AST, ALP, triglyceride, LDL, and cholesterol, as well as a decreased levels of HDL. These findings suggest that individuals with MAFLD are at greater risk for abnormalities in liver activity and dyslipidemia. ALT, AST, and GGT are three hepatic enzymology indices extensively utilized in medical research. Hepatic steatosis and pathological injury can occur due to excessive accumulation of fat in liver cells, leading to elevated levels of ALT, AST, and GGT. MetS, characterized by increased triglyceride, lowered HDL, and a greater prevalence of hypertension and diabetes, is closely linked to MAFLD.^33^

 One of the main metabolic indicators and a contributing factor to MAFLD is being overweight. A meta-analysis conducted in Europe, the United States, and Asia, included 11 separate groups. The research found that obesity significantly increases the risk of developing hepatocellular carcinoma.^34^ However, there is also evidence that some individuals with MAFLD who have significant abnormalities in their metabolic profiles such as lipids or glucose, do not exhibit obesity (lean MAFLD).^35^ Consistent with this, our data indicated that there was a significant relationship between obesity and MAFLD.

 Yuan et al discovered that the prevalence of MAFLD increased with age in both men and women (*P* < 0.001), and MAFLD was more common in men (36.80% vs. 28.65%). They hypothesized that the disparity is due to hormonal effects.^36^ However, our findings contradict this, suggesting that being female is actually a significant factor in predicting the occurrence of MAFLD. A higher rate of obesity is observed in our female population, contributing to the increased prevalence of MAFLD.

 The impact of alcohol use on MAFLD remains uncertain thus far. Prior research indicates that alcohol use may have either a positive or negative correlation with MAFLD in comparison to abstaining from alcohol.^37-40^ However, our investigation discovered no connection between drinking alcohol and the prevalence of MAFLD, which aligns with the findings of Yuan et al.^36,41,42^ Alcohol use is prohibited in Iran and only 5% of the PCS population have reported its use. Therefore, this might have been under-estimated in our study.

 Genetics plays an important role in the development of MAFLD. Limited studies have been conducted on the heritability of hepatic fibrosis and steatosis, and the genetic factors linked to these conditions are not well comprehended.^18^ It is necessary to identify the genetic factors associated with hepatic fibrosis and steatosis, especially in adult populations and those who have lean MAFLD.^19,20^ Despite advances in our understanding of the genetic factors contributing to MAFLD, there is still much to learn about the underlying mechanisms and how to effectively manage this disease

 The data for this study was extracted from a large population with diverse ethnicities, a large sample size and potential confounders were adjusted for through the availability of extensive clinical data. Also our study was a retrospective cohort study. Generally, cohort studies have a higher level of evidence compared to other observational studies. However, the study also had some limitations. First, MAFLD was diagnosed by ultrasonography, which is suggested as an initial-line imaging technique for MAFLD by the Association for the Study of the Liver.^43^ However, ultrasonography is less sensitive than biopsy, which is considered the gold standard, for diagnosing mild hepatic steatosis.^44^ Therefore, the actual prevalence of MAFLD might have been higher than reported. As a result, there may have been some variables that could be risk factors for MAFLD that we were unable to identify due to the underestimation of MAFLD. Second, alcohol use is prohibited in Iran and self-reports may underestimate its actual use. Despite the diverse ethnicities in our population, it is important to consider cultural and geographical variations in risk factors and disease prevalence should be considered when generalizing our finding to a broader population.

## Conclusion

 In conclusion, our study found that consuming opium decreases the likelihood of MAFLD in CAD patients, since these patients have decreased appetite and lower body mass index (BMI). On the other hand, female gender, having diabetes, high waist circumference, high triglyceride levels, and high ALT levels increase the probability of MAFLD in CAD patients.
